# Genome assemblies for two Neotropical trees: *Jacaranda copaia* and *Handroanthus guayacan*

**DOI:** 10.1093/g3journal/jkab010

**Published:** 2021-01-28

**Authors:** John T Burley, James R Kellner, Stephen P Hubbell, Brant C Faircloth

**Affiliations:** 1 Department of Ecology and Evolutionary Biology, Brown University, Providence, RI 02912, USA; 2 Institute at Brown for Environment and Society, Brown University, Providence, RI 02912, USA; 3 Department of Ecology and Evolutionary Biology, University of California—Los Angeles, Los Angeles, CA 90095, USA; 4 Department of Biological Sciences and Museum of Natural Science, Louisiana State University, Baton Rouge, LA 70803, USA

**Keywords:** genome assembly, tropical tree, Chicago library, dovetail, *Jacaranda copaia*, *Handroanthus* (*Tabebuia*) *guayacan*

## Abstract

The lack of genomic resources for tropical canopy trees is impeding several research avenues in tropical forest biology. We present genome assemblies for two Neotropical hardwood species, *Jacaranda copaia* and *Handroanthus* (formerly *Tabebuia*) *guayacan*, that are model systems for research on tropical tree demography and flowering phenology*.* For each species, we combined Illumina short-read data with *in vitro* proximity-ligation (Chicago) libraries to generate an assembly. For *Jacaranda copaia*, we obtained 104X physical coverage and produced an assembly with N50/N90 scaffold lengths of 1.020/0.277 Mbp. For *H. guayacan*, we obtained 129X coverage and produced an assembly with N50/N90 scaffold lengths of 0.795/0.165 Mbp. *J. copaia* and *H. guayacan* assemblies contained 95.8% and 87.9% of benchmarking orthologs, although they constituted only 77.1% and 66.7% of the estimated genome sizes of 799 and 512 Mbp, respectively. These differences were potentially due to high repetitive sequence content (>59.31% and 45.59%) and high heterozygosity (0.5% and 0.8%) in each species. Finally, we compared each new assembly to a previously sequenced genome for *Handroanthus impetiginosus* using whole-genome alignment. This analysis indicated extensive gene duplication in *H. impetiginosus* since its divergence from *H. guayacan*.

## Introduction

Although there is a fruitful history of population genetic and phylogenetic research on tropical forest trees using allozymes, microsatellite loci, and chloroplast loci (Hamrick and Murawski 1991; Lowe 2005; Lohmann 2006; Olmstead *et al.* 2009; Ragsac *et al.* 2019) modern genomic approaches have rarely been used to study these communities (c.f. Collevatti *et al.* 2019; Brousseau *et al.* 2020). As a result, tropical forest trees are under-represented in the universe of plant genomic studies (Plomion *et al.* 2016; Fetter *et al.* 2017), aside from a few commercially important species. However, these types of studies would advance our understanding of tropical forest communities—the ecological dynamics of which have been studied for decades (Hubbell 1979; Phillips *et al.* 1994; Laurance *et al.* 2018). For example, genome-enabled studies of tropical trees would allow us investigate: the origin and maintenance of tropical forest diversity (Dick and Pennington 2019), the genetic basis of tropical tree traits (Wright *et al.* 2010), or the potential for evolutionary responses of tree populations to climate change and deforestation (Hamrick 2004; Phillips *et al.* 2008; Alberto *et al.* 2013). These efforts, and more (Antonelli *et al.* 2018), will accelerate with the sequencing and assembly of additional tropical tree reference genomes by individual research groups and larger collaborative efforts like the 10KP project (Cheng *et al.* 2018).

**Figure 1:**
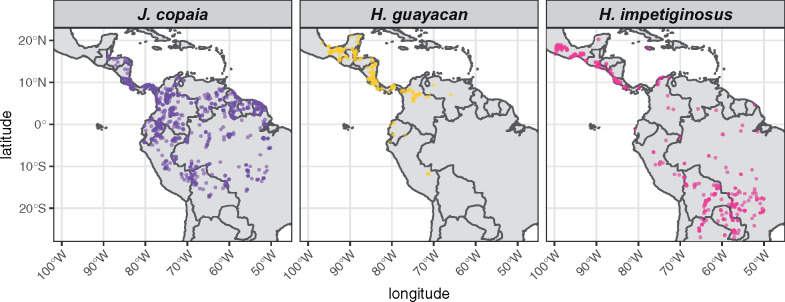
Geographic distributions of herbarium records for species sequenced in this study (*J. copaia* and *H. guayacan*) as well as the previously sequenced *H. impetiginosus* (Silva-Junior *et al.* 2018). Data obtained from gbif.org.

We therefore developed reference genomes for *Jacaranda copaia* and *Handroanthus guayacan* (hereafter JACO and HAGU). Both species are insect-pollinated, wind-dispersed, predominately outcrossing canopy trees that are widely distributed throughout the Neotropics (Gentry 1974, 1976, 1992; Condit *et al.* 2011; Figure 1). *Jacaranda* is the sister clade to the core Bignoniaceae, a family that includes 827 recognized species of trees, shrubs, and lianas (Olmstead *et al.* 2009) with an estimated divergence of approximately 40–60 million years ago (Wikström *et al.* 2001; Magallón *et al.* 2015). JACO, HAGU, and their close relatives are important study systems for understanding tropical tree population dynamics (Jones and Hubbell 2006; Jones and Comita 2008; Kellner and Hubbell 2017, 2018), the diversification and proximate cues of flowering phenology (Gentry 1974; Reich and Borchert 1982; Lobo-Segura 2019), Neotropical phylogeography (Collevatti *et al.* 2012, 2019; Scotti-Saintagne *et al.* 2013; Vitorino *et al.* 2018), late-acting reproductive self-incompatibility (Bittencourt Júnior 2017), and genome size evolution (Collevatti and Dornelas 2016). In addition, many tree species in this group are economically important and simultaneously of conservation concern due to legal and illegal logging (Schulze *et al.* 2008; Brancalion *et al.* 2018), creating a need for the development of molecular markers for species identification and timber tracking (Meyer-Sand *et al.* 2018; Sebbenn *et al.* 2019). Although important for addressing these types of questions, genomic approaches have rarely featured in these investigations, with the exception of population genomic surveys of *H. impetiginosus* (hereafter HAIM) (Collevatti *et al.* 2019), which were facilitated by a reference assembly produced for that species (Silva-Junior *et al.* 2018).

**Figure 2:**
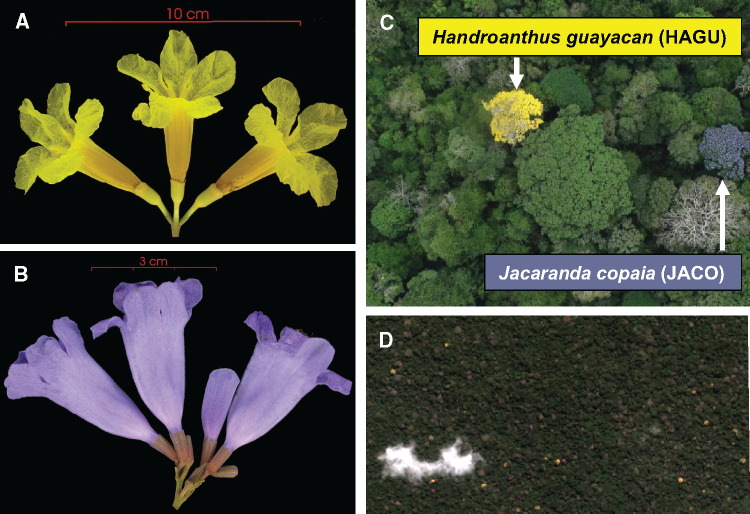
Flowers of (A) *H. guayacan* (HAGU) and (B) *J. copaia* (JACO)*.* While in bloom, individual tree crowns are highly conspicuous in (C) aerial and (D) satellite imagery of a lowland tropical forest in Central Panama. Image credits: (A and B) Steven Paton, Smithsonian Tropical Research Institute (STRI); (C) Jonathan Dandois, STRI.

Our primary reason for sequencing JACO and HAGU genomes was to create opportunities for combining population genomic analyses with estimates of demographic rates and phenotypic variation. This possibility results from highly conspicuous flowering in each species (Figure 2). Individual trees can be mapped and monitored using high-resolution satellite data, particularly among species in the genus *Handroanthus* (Kellner and Hubbell 2017, 2018; Kellner *et al.* 2019). Using satellite data to observe individuals while flowering also produces an unambiguous indicator of reproductive status and population variation in phenological traits across large areas.

In this study, we describe the sequencing and assembly of reference genomes for JACO and HAGU, we summarize the basic genomic characteristics of each species estimated using our assemblies, and we use whole genome alignment to compare both JACO and HAGU assemblies with the HAIM genome announced by Silva-Junior *et al.* (2018).

## Materials and methods

### Tissue collection from vouchered specimens

We sampled JACO tissue from one individual grown from seed in the Brown University Plant Environmental Center using seeds we purchased commercially that were collected in Huila, Colombia during November 2016. We germinated seeds in Sungro propagation mix in an irrigation tent at 27°C, and we transferred individuals to larger pots as they grew. After approximately 12 months, we collected fresh leaf tissue from one individual. Tissue was flash-frozen in liquid nitrogen and then transferred to –80°C storage within 30 min. All tissue was stored at −80°C until shipment. We archived a voucher specimen from the sampled individual in the Brown University Herbarium (accession P BRU 00008453).

For HAGU, we collected leaf tissue directly from a vouchered adult individual (accession HBG# 8421) at the Foster Botanical Garden in Honolulu, Hawai’i. Leaf tissue was flash-frozen in liquid nitrogen and then stored at −80°C until shipment. The individual we sampled was originally collected from a wild population of *H. guayacan* in the Canal Zone Experimental Gardens, Panama during 1939 and is now a large tree. Herbarium vouchers for this individual are stored in the Bishop Museum in Honolulu, Hawai’i (accession IDs BISH 43029, BISH 43030, BISH 43031).

### Preliminary *de novo* assemblies using short-insert read libraries and the Meraculous assembler

We shipped frozen leaf tissue on dry ice to Dovetail Genomics, LLC (Scotts Valley, CA), for DNA extraction and library preparation. Dovetail staff extracted DNA using a standard CTAB DNA extraction, and extracts were sheared for Illumina library preparation using a Bioruptor Pico. For each species, Dovetail staff prepared two short-insert libraries with insert lengths of approximately 400 and 500 bp, following the Illumina TruSeq PCR-free protocol with no deviations. To validate these libraries, Dovetail staff used an Illumina MiSeq to generate several million paired-end (PE), 250 bp reads from each species.

Although both species had been identified by expert botanists prior to sequencing, *Jacaranda* and *Handroanthus* species can be difficult to identify using morphology. To validate the species identity of both extracts/libraries prior to sequencing them to depth, we assembled the reads generated for library validation using Spades (Bankevich *et al.* 2012) and a k-mer length of 55. Because chloroplast DNA was at high concentrations in both libraries, this enabled us to assemble large segments of the chloroplast genome (Straub *et al.* 2012). We used the NCBI BLAST (Altschul *et al.* 1990) search tool with no query limits to find the closest matches of our contigs to published sequences in GenBank. For JACO, the chloroplast DNA contigs matched more closely with *J. copaia* chloroplast sequences than for any other *Jacaranda* species available in GenBank. For HAGU, bioinformatic species validation was more complicated because few full cpDNA assemblies are available for *Tabebuia* and *Handroanthus* species. We overcame this difficulty by extracting standard barcoding genes (matK, rbcL, and psbA) (Kress *et al.* 2009), and BLASTing these against the GenBank dataset, representing 8 *Handroanthus* species. For matK and psbA, the top hits to our HAGU query were *H. guayacan* isolates (including one voucher specimen) with 100% identity over 100% length. For rbcL, however, isolates from *H. albus* and *H. guayacan* both matched with 100% identity over 100% length. Based on these three markers, given that *H. albus* does not occur where our sample was collected in Central Panama (Gentry 1992), and because our sample is from a vouchered specimen (identified by Alwyn Gentry), we are confident in the species identification for HAGU. After species validation, all libraries were sequenced using PE, 150 bp sequencing on an Illumina NextSeq 500.

Following sequencing, reads were trimmed to remove sequencing adapters and low-quality bases using Trimmomatic (Bolger *et al.* 2014). Dovetail staff profiled short-insert reads at a variety of k-mer values (19, 31, 49, 55, 79, and 109) to estimate genome size, heterozygosity, and repeat content. To optimize coverage and achieve a balance between repetitive and heterozygous fractions during assembly, Dovetail staff fit negative binomial models to the k-mer size distributions. These models suggested that a k-mer size of 55 for both species and homozygous peak depths of 78.0 (JACO) and 162.0 (HAGU) were optimal for the Meraculous assembler. Dovetail staff generated *de novo* assemblies by inputting paired-end libraries (described above) for each species to Meraculous v2 (Chapman *et al.* 2011) with a k-mer size of 55 and minimum k-mer frequencies of 15 (JACO) and 29 (HAGU). Based on the level of heterozygosity in both genomes, Dovetail staff used the pseudo-haploid mode in Meraculous.

### Assembly improvement using Chicago read libraries and the HiRise scaffolding pipeline

Following *de novo* assembly with Meraculous, Dovetail staff prepared proprietary “Chicago” libraries following the methods described in Putnam *et al.* (2016). Briefly, they reconstituted ∼500 ng of HMW genomic DNA [mean fragment length = 60 kb (HAGU)], into chromatin *in vitro* and fixed the reconstituted DNA with formaldehyde. Then, they digested fixed chromatin with DpnII, filled in 5’ overhangs with biotinylated nucleotides, and ligated free, blunt ends. After ligation, they reversed crosslinks and purified the DNA from protein. Dovetail staff treated purified DNA to remove biotin that was not internal to ligated fragments and sheared the resulting DNA to ∼350 bp mean fragment size using a Bioruptor Pico. Dovetail staff then prepared sequencing libraries from these sheared DNA using NEBNext Ultra enzymes (New England Biolabs, Inc.) and Illumina-compatible adapters. They isolated biotin-containing fragments using streptavidin beads before PCR enrichment of each library. Dovetail Staff then sequenced amplified libraries on an Illumina HiSeq X Ten platform using PE 150 reads. Dovetail staff combined preliminary *de novo* Meraculous assemblies along with shotgun reads and Chicago read libraries and used the HiRise scaffolding software (Putnam *et al.* 2016) to iteratively break and reassemble contigs into scaffolds based on long-range linkage information provided by the Chicago reads.

### Assessment of assembly contiguity and completeness

We used NX and LX statistics to evaluate assembly contiguity, and we estimated assembly completeness using BUSCO v3.0.2 (Simão *et al.* 2015) to search each assembly for a set of 2121 eudicot Benchmarking Universal Single-Copy Orthologs (BUSCOs) (odb10). We performed these analyses (and analyses described below) for our JACO and HAGU assemblies, as well as the previous published genome assembly of HAIM (GCA_002762385.1) (Silva-Junior *et al.* 2018). Finally, we used Pseudohaploid (https://github.com/schatzlab/pseudohaploid) to identify redundant sequence that may exist in HiRise assemblies due to uncollapsed heterozygous haplotypes.

### Identification and classification of repetitive elements

We used the RepeatModeler v.1.0.11 (Smit and Hubley 2008) and RepeatMasker v.4.0.7 (Smit *et al.* 2013) to identify and classify repetitive DNA in the genome assemblies. RepeatModeler first builds a database of putative repeats from an NCBI database search guided by the input assembly, then employs two *de novo* repeat finding programs [RECON v.1.08 (Bao and Eddy 2002) and RepeatScout v.1.0.5 (Price *et al.* 2005)] to refine and classify models of putative interspersed repeats. RepeatMasker then screens the input assembly for putative repeat elements and provides summaries of genome-wide repeat content.

### Genome alignment

To compare our JACO and HAGU assemblies to the HAIM assembly (Silva-Junior *et al.* 2018), we performed whole genome alignments using the NUCmer module of MUMmer v.4.0.0beta2 (Marçais *et al.* 2018) with default parameters, and we summarized the number and fraction of aligned scaffolds and bases, as well as the lengths and identity score of alignments, using the dnadiff module of MUMmer. In each species comparison, we used the assembly with the highest N50 and N90 as the reference sequence and the other assembly as the query. We also performed an alignment of JACO and the high-quality assembly of *Olea europeae* (Cruz *et al.* 2016), which is another species within the taxonomic order (Lamiales) that includes JACO and HAGU.

### Data availability

All sequencing data and assemblies are available through NCBI BioProject PRJNA614908. The Whole Genome Shotgun assemblies have been deposited at DDBJ/ENA/GenBank under the accession JACGDI000000000 (JACO) & JACFYI000000000 (HAGU). The versions described in this manuscript are JACGDI010000000 and JACFYI010000000. Supplemental material is available at figshare: https://doi.org/10.25387/g3.12964592.

## Results and discussion

### The *Jacaranda copaia* genome

Sequencing of two paired-end libraries with mean inserts of length 413 and 519 bp produced a total of 369.8 million read pairs (Supplementary Table S1). Dovetail staff estimated a genome size of 799 Mbp based on k-mer analysis of these reads using the optimal k-mer length of 55 bp; as well as heterozygosity of 0.5% and repeat content of 18.6% (Supplementary Table S2). This genome size estimate is within the range of estimates for three other *Jacaranda* species obtained via flow cytometry (558.6–1274.0 Mbp) (Collevatti and Dornelas 2016). The preliminary assembly produced by Meraculous contained 53,440 contigs with N50 of 36.3 Kbp (Table 1). These contigs were joined by Meraculous into 29,490 scaffolds with a total length of 613.9 Mbp, an N50 of 56.7 Kbp, and L90 of 12,202 scaffolds (Table 1).

**Table 1:** Basic genome assembly metrics and quality statistics

Species	*J. copaia*		*H. guayacan*	
Assembly method	Meraculous	Dovetail HiRise assembly	Meraculous	Dovetail HiRise assembly
**Total length**	613.86 Mbp	616.19 Mbp	336.39 Mbp	339.77 Mbp
**Scaffold N50**	0.057 Mbp	1.020 Mbp	0.017 Mbp	0.795 Mbp
**Scaffold N90**	0.010 Mbp	0.277 Mbp	0.004 Mbp	0.165 Mbp
**Scaffold L50**	2,974 scaffolds	184 scaffolds	5,519 scaffolds	113 scaffolds
**Scaffold L90**	12,202 scaffolds	624 scaffolds	21,878 scaffolds	452 scaffolds
**Longest scaffold**	506,509 bp	5,337,482 bp	148,601 bp	4,933,827 bp
**Number of scaffolds**	29,490	5,869	38,393	3,857
**Number of scaffolds >1 kb**	29,490	5,869	38,393	3,856
**Number of scaffolds >10 kb**	12,474	1,086	10,322	825
**Proportion of assembly in scaffolds >10 kb**	0.90	0.98	0.69	0.98
**Number of contigs**	53,440	53,041	70,867	70,146
**Contig N50**	36.27 Kbp	36.74 Kbp	12.25 Kbp	12.47 Kbp
**GC content**	—	32.99%	—	33.24%
**Number of gaps**	23,950	47,236	32,474	66,288
**Percent of genome in gaps**	0.44%	0.81%	1.32%	2.30%

We then sequenced two Chicago libraries, which yielded 421 million read pairs (Supplementary Table S1) or 104.00X physical coverage of the Meraculous assembly. HiRise made 23,645 joins and 24 breaks to the Meraculous assembly, closing 359 gaps to form the Chicago assembly. The improved JACO HiRise assembly contained 5869 scaffolds with a total length of 616.19 Mbp, or 77.1% of the k-mer estimated genome size of 799 Mbp (Table 1, Supplementary Table S2); and it had an N50 of 1.020 Mbp, an L90 of 624 scaffolds, and a longest scaffold of 5.34 Mbp (Table 1).

### The *Handroanthus guayacan* genome

Sequencing of two paired-end libraries with mean inserts of length 414 and 518 bp produced a total of 558.78 million read pairs (Supplementary Table S1). Dovetail staff estimated a genome size of 512 Mbp based on k-mer analysis of these reads using the optimal k-mer length of 55 bp; as well as heterozygosity of 0.8% and repeat content of 19.24% (Supplementary Table S2). The HAGU genome size estimate is within the range of estimates for seven other *Handroanthus* species obtained via flow cytometry (Collevatti and Dornelas 2016). The preliminary assembly produced by Meraculous contained 70,867 contigs with N50 of 12.2 Kbp (Table 1). These contigs were joined by Meraculous into 38,393 scaffolds with a total length of 613.9 Mbp, an N50 of 17.1 Kbp, and an L90 of 21,878 scaffolds (Table 1).

We then sequenced one Chicago library, which yielded 414 million read pairs (Supplementary Table S1), or 128.99X physical coverage of the Meraculous assembly. HiRise made 34,560 joins and 24 breaks to the Meraculous assembly, closing 746 gaps to form the Chicago assembly. The JACO HiRise assembly contained 3857 scaffolds with a total length of 339.77 Mbp, or 66.4% of the k-mer estimated genome size of 512 Mbp (Table 1, Supplementary Table S2); and it had an N50 of 0.795 Mbp, an L90 of 452 scaffolds, and a longest scaffold of 4.93 Mbp (Table 1).

### Assembly quality assessments

Chicago scaffolding of the Meraculous assembly improved assembly contiguity for both species, illustrated by 17.9-fold (JACO) and 46.8-fold (HAGU) increase in scaffold N50 (Table 1). For both species, 98% of HiRise assemblies were contained in scaffolds longer than 10 Kbp (Table 1).

Improvements to the Meraculous assembly by Chicago scaffolding were also evident by fewer missing and fragmented BUSCOs for both species. Complete and unfragmented BUSCOs increased from 92.3% to 95.8% (JACO) and 82.8% to 87.9% (HAGU) as a result of HiRise scaffolding (Table 2). For comparison to our BUSCO results, we also analyzed the HAIM assembly, which showed a similar level of BUSCO completeness to HAGU despite different assembly methods and contiguity statistics (Table 2).

**Table 2:** Genome completeness assessed using 2,121 eudicot Benchmarking Universal Single-Copy Orthologs (BUSCOs)

Species:	*J. copaia*		*H. guayacan*		*H. impetiginosus*
Assembly:	Meraculous	HiRise	Meraculous	HiRise	Himp0.1
**Complete**	1,957	2,032	1,754	1,864	1,847
**Single copy**	1,844	1,911	1,676	1,775	1,574
**Duplicated**	113	121	78	89	273
**Fragmented**	82	34	179	99	103
**Missing**	82	55	188	158	171
**% Complete**	**92.3**	**95.8**	**82.8**	**87.9**	**87.1**

Despite the high level of genic content represented in each assembly, both comprised a relatively low fraction of their estimated genome size, compared to other draft genome assemblies of trees (Silva-Junior *et al.* 2018; Supplementary Table S3). This may be a product of the assembly algorithm, Meraculous, which will not make ambiguous joins and, therefore, is unlikely to resolve repeat-dense regions (Chapman *et al.* 2011). If this is the case, the relatively high BUSCO scores for both species suggest that the difference between estimated and realized genome size may result from the extensive presence of repetitive sequence.

### Repetitive sequences

Although Meraculous will not resolve some repeat-dense regions, we used standard procedures to identify and classify the repetitive elements in both JACO and HAGU assemblies, and we compared these results to estimates for HAIM (Silva-Junior *et al.* 2018). We identified 59.31% (JACO) and 45.59% (HAGU) total repetitive sequence content in each of our assemblies (Table 3). The vast majority of repeats were interspersed, of which approximately one third were not further classified (Table 3). Of the interspersed repeats that were classified, <1% were long interspersed nuclear elements (LINEs), 24.93% in JACO and 10.85% in HAGU were long terminal repeats (LTRs), and 2.97% in JACO and 1.53% in HAGU were DNA elements (Table 3). HAGU and HAIM contained similar repetitive sequence fractions in almost all categories, relative to JACO (Table 3).

**Table 3:** Predicted repetitive sequence content in assemblies

	*J. copaia*			*H. guayacan*			*H. impetiginosus*		
	Number of elements	Length (Mb)	Fraction of assembly (%)	Number of elements	Length (Mb)	Fraction of assembly (%)	Number of elements	Length (Mb)	Fraction of assembly (%)
**Total interspersed repeats**	619,981	356.7	57.89	451,277	149.8	44.08	634,428	231.8	45.06
**LINEs**	3,759	3.3	0.54	3,081	2.1	0.63	3,974	3.3	0.66
**LTR elements**	105,619	153.6	24.93	43,361	36.9	10.85	52,006	56.7	11.26
**DNA elements**	27,586	17.2	2.97	9,425	5.2	1.53	18,240	10.1	2.01
**Unclassified**	483,017	182.6	29.63	395,410	105.6	31.07	560,208	161.7	32.13
**Simple repeats**	151,890	9.0	1.47	125,684	5.1	1.51	182,721	7.8	1.54
**Low complexity**	26,279	1.3	0.21	23,734	1.1	0.33	33,613	1.6	0.33
**Total**	**798,150**	**367.0**	**59.57**	**600,695**	**156.0**	**45.92**	**850,762**	**241.2**	**46.93**

Although repeat fractions estimated by RepeatMasker are more reliable than those estimated using k-mer analysis, the proportions of genome-wide repeat content and individual repeat classes we computed are likely underestimated. This is because (1) both assemblies are incomplete with respect to estimated genome sizes (see above) and (2) the assembly technology used is systematically biased to perform poorly for genome regions that contain long-repeats (Chapman *et al.* 2011) or that are highly repetitive, such as heterochromatic regions (Altemose *et al.* 2014). Given that LTR elements typically comprise the majority of repetitive sequence in plant genomes (Vitte and Bennetzen 2006; Tenaillon *et al.* 2011), the high-estimated fraction of LTR repeats helps explain the difference we observed between estimated genome size and realized assembly length.

### Genome alignments

Because we were interested in: (1) obtaining a broad comparison of sequence divergence among the taxa, (2) gauging the extent of possible chromosomal rearrangements (specifically and duplications), and (3) identifying potential assembly errors, we performed whole-genome sequence alignments between the JACO, HAGU, and HAIM genome assemblies.

**Figure 3:**
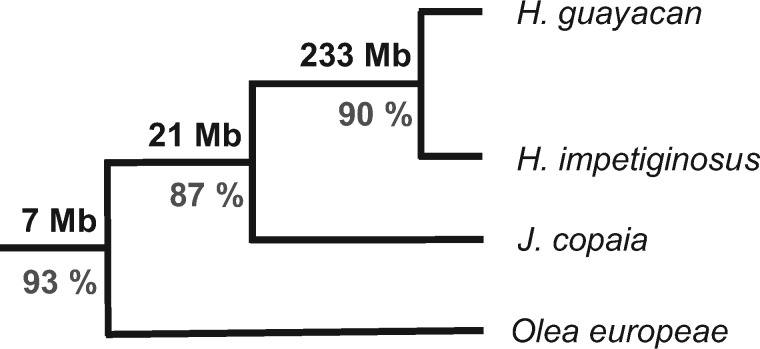
Depiction of relationships among the three currently available Bignoniaceae genomes and a high-quality assembly of the European olive tree (*Olea europeae*) derived from relationships of *Jacaranda* and *Handroanthus* resolved in Grose and Olmstead (2007a) and Olmstead *et al.* (2009). Node values depict whole genome alignment summary metrics: total length of 1-to-1 alignments (black) and average percentage identify of 1:1 alignments (gray).

Assemblies for HAGU and HAIM shared a high fraction of aligned scaffolds: 3655 (94.76%) for HAGU and 12,398 (93.90%) for HAIM (Table 4). This alignment includes 233 Mbp in 149,208 1-to-1 best alignments—locations where HAGU aligned to its best hit in HAIM, and vice versa—with an average identity of 90.34%, which represents approximately 68.6% of the HAGU assembly (total length of 339.77 Mbp) and 46.3% of HAIM (total length of 503.29 Mbp) (Table 4; Figure 3). In contrast to the HAGU-HAIM comparison, JACO and HAGU have a far less extensive alignment, with only 22 Mbp of both genomes in 68,689 1-to-1 best alignments, although the average identity of those alignments is high (88.73%); and a similar result was obtained in the JACO-HAIM alignment (Table 4; Figure 3). This low degree of alignment likely reflects divergence of almost all but the most highly conserved sequence between *Handroanthus* and *Jacaranda*, which is unsurprising given that they shared a common ancestor >40 million years ago (Lohmann *et al.* 2013; Magallón *et al.* 2015).

**Table 4:** Genome alignment summary statistics

Species comparison	*H*. *guayacan* and *H*. *impetiginosus*		*J*. *copaia* and *H*. *impetiginosus*		*J*. *copaia* and *H*. *guayacan*	
	Reference	Query	Reference	Query	Reference	Query
	HAGU	HIMP	JACO	HIMP	JACO	HAGU
**Sequences**	—	—	—	—	—	—
**Total**	3,857	13,204	5,869	13,204	5,869	3,857
**Aligned**	3,655 (94.76%)	12,398 (93.90%)	2,172 (37.01%)	8,150 (61.72%)	2,257 (38.46%)	1,937 (50.22%)
**Unaligned**	202 (5.24%)	806 (6.10%)	3,697 (62.99%)	5,054 (38.28%)	3,612 (61.54%)	1,920 (49.78%)
**Bases**	—	—	—	—	—	—
**Total (Mb)**	339.77	503.29	616.19	503.29	616.19	339.77
**Aligned (Mb)**	266.8 (78.52%)	374.8 (74.46%)	21.8 (3.54%)	32.8 (6.52%)	23.8 (3.86%)	26.8 (7.87%)
**Unaligned (Mb)**	72.9 (21.48%)	128.5 (25.54%)	594.4 (96.46%)	470.5 (93.48%)	592.4 (96.14%)	313.0 (92.13%)
**Alignments**	—	—	—	—	—	—
**1-to-1**	149,208	149,208	44,485	44,485	68,689	68,689
**Total length (Mb)**	232.8	232.7	20.2	20.1	22.2	22.2
**Av. length (Bp)**	1561	1560	454	453	324	323
**Av. identity (%)**	90.34	90.34	87.27	87.27	88.73	88.73
**Many-to-Many**	447,736	447,736	91,126	91,126	174,083	174,083
**Total length (Mb)**	479.4	479.1	40.8	40.7	38.3	38.2
**Av. length (Bp)**	1071	1070	448	447	220	220
**Av. identity (%)**	89.94	89.94	91.87	91.87	92.64	92.64

**Figure 4:**
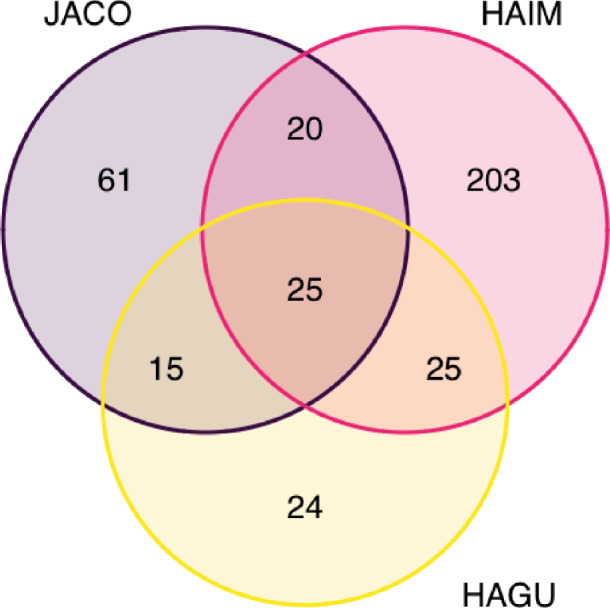
Shared and unique duplicated BUSCOs in genome assemblies for JACO, HAGU, and HAIM.

Further inspection of HAGU-HAIM alignments suggests the possibility of extensive gene duplication since their divergence. The length of many-to-many alignments between HAGU and HAIM represents 141% of the HAGU assembly and 95% of HAIM (Table 4). Therefore, the length of the HAGU-HAIM many-to-many alignments exceeds the HAGU assembly by 139 Mbp. One possibility to explain this excess is that many gene duplications occurred in HAIM since its divergence from a common ancestor shared with HAGU such that multiple gene copies in HAIM are homologous with, and relatively undifferentiated from, single-copy regions in HAGU. Another possibility is that the HAIM assembly contains a relatively high proportion of uncollapsed heterozygous sequence. This can occur during genome assembly if highly heterozygous segments in a diploid genome are mistakenly characterized as two separate sequences rather than homologous alleles on opposite chromosome arms, thereby erroneously extending the length of a genome assembly. To investigate these two possibilities, we first generated a Venn diagram of BUSCOs obtained in each assembly. HAIM has 203 unique duplicated BUSCOs compared to 24 in HAGU (Figure 4); and the total number of duplicated BUSCOs is threefold higher in HAIM than HAGU (Table 2). Most of the duplicated BUSCOs in HAIM appear as single copies in HAGU (71%); but the opposite is not true with only 42% of HAGU duplicated BUSCOs appearing as single copies in HAIM. While this result is consistent with the hypothesis that HAIM accumulated numerous duplications since its divergence from the HAIM-HAGU common ancestor, it does not exclude the possibility of assembly artifact. However, Pseudohaploid identified only 27.5 Mbp of probable uncollapsed heterozygous sequence in HAIM (and none in HAGU or JACO). In addition, the HAIM assembly length (503 Mbp) is close to the genome size estimated through flow cytometry for that species (499.8 Mbp) (Collevatti and Dornelas 2016; Silva-Junior *et al.* 2018). It therefore seems unlikely that uncollapsed heterozygous sequences account for the excess of many-to-many alignments between HAGU-HAIM. In sum, this analysis suggests gene duplications may have occurred extensively in HAIM since its divergence from a common ancestor shared with HAGU, although we cannot rule out the possibility of gene-loss in HAGU during the same period.

### Outlook

The genome assemblies for JACO and HAGU presented here meet standards of the field for use in studies involving genetic marker discovery, gene and gene family evolution, comparative genomics, phylogenomics and ecological genomics (Sork *et al.* 2016; Plomion *et al. 2018;* Tuskan *et al.* 2018) (Supplementary Table S3). These assemblies add to a rapidly expanding collection of publicly available genomic resources for the Bignoniaceae including, to date, six transcriptomes (Leebens-Mack *et al.* 2019; Zhang *et al.* 2020) and the HAIM genome assembly (Silva-Junior *et al.* 2018). Each assembly may be further improved by the incorporation of long-reads for gap-filling (English *et al.* 2012; Rhie *et al.* 2020), linkage maps created by population resequencing (The Heliconius Genome Consortium 2012; Kawakami *et al.* 2017), or syntenic information from alignments of multiple draft Bignoniaceae genomes (Kolmogorov *et al.* 2018). Chromosome-scale assemblies of additional Bignoniaceae will aid further investigations of the remarkable variation in genome size and chromosome number documented in these taxa (Collevatti and Dornelas 2016; Mendes *et al.* 2018). Furthermore, phylogenomic investigations of *Handroanthus* and relatives in the Tabebuia alliance will increase resolution of unclear speciation histories (Grose and Olmstead 2007a, 2007b) that may involve hybridization among sympatric species (Gentry 1992; Pinheiro *et al.* 2018). Finally, reference genomes for JACO and HAGU will facilitate ongoing research efforts to investigate landscape-scale interactions between population genetic processes and population dynamics of tropical canopy trees where genome resequencing data are combined with high throughput surveys of individual-level traits, such as flowering time, and population demographic parameters such as density, recruitment and mortality (Kellner and Hubbell 2017, 2018).
